# Secreted AZGP1 induced by 5-FU binds to PD-L1 and promotes apoptosis in cholangiocarcinoma

**DOI:** 10.1186/s10020-025-01362-8

**Published:** 2025-09-26

**Authors:** Ji-Eun You, Do Yeon Kim, Hyeseon Yun, Dong-In Koh, Yea Seong Ryu, Dong-Hoon Jin

**Affiliations:** 1 Asan Institute for Life Science, Asan Medical Center, Seoul, Republic of Korea; 2 Department of Pharmacology, Asan Medical Center, University of Ulsan College of Medicine, Seoul, South Korea; 3 Department of Convergence Medicine, Asan Institute for Life Science, Asan Medical Center, Seoul, South Korea; 4 Department of Pharmacology, University of Ulsan College of Medicine, Seoul, Republic of Korea; 5 Department of Convergence Medicine and Pharmacology, Asan Medical Center, University of Ulsan College of Medicine, 88 Olympicro-43gil, Songpa-gu, Seoul, Republic of Korea

**Keywords:** Cholangiocarcinoma, Soluble AZGP1, PD-L1, 5-FU

## Abstract

**Background:**

Cholangiocarcinoma (CCA) is an aggressive malignancy with limited treatment options and poor clinical outcomes. The identification of predictive biomarkers that can enhance chemotherapy efficacy and inform immune modulation remains an urgent unmet need.

**Methods:**

We investigated the biological role and regulatory mechanisms of soluble AZGP1 in CCA using western blotting, ELISA, flow cytometry, and *in vitro/in vivo* functional assays. A combination of pharmacological treatments and gene expression analyses was employed to explore pathway dynamics and immune responses.

**Results:**

We found that soluble AZGP1, which is typically downregulated in CCA, is significantly upregulated following 5-fluorouracil (5-FU) treatment. Mechanistically, 5-FU modulates the AKT–Foxo1 signaling pathway, promoting nuclear translocation of Foxo1 and activation of AZGP1. Elevated soluble AZGP1 levels were shown to interact with PD-L1, enhancing apoptosis in cancer cells. Analyses of peripheral blood mononuclear cells (PBMCs) further confirmed the immune-modulating potential of AZGP1 in the tumor microenvironment.

**Conclusions:**

Our findings reveal that soluble AZGP1 acts as a predictive biomarker for 5-FU efficacy in cholangiocarcinoma and exerts pro-apoptotic and immunomodulatory functions via interaction with PD-L1. These insights may contribute to the development of more precise and effective therapeutic strategies in CCA treatment.

**Supplementary Information:**

The online version contains supplementary material available at 10.1186/s10020-025-01362-8.

## Background

Cholangiocarcinoma (CCA) is a highly aggressive neoplasm that originates from the biliary tract [Elvevi et al. 2022]. The surgical management of CCA presents significant challenges when compared to other carcinoma types, primarily due to its histological characteristics, including its anatomical positioning and proximity to surrounding organs [Elvevi et al. 2022; Brindley 2021]. These factors contribute to a dismal prognosis, marked by elevated mortality rates and frequent recurrences [Banales et al. 2016; Andersen et al. 2012; Vogel 2014], which are further exacerbated by the insidious progression of the disease, difficulties in early detection, and a limited array of therapeutic options. A thorough review of retrospective studies has shaped the clinical practice guidelines promulgated by the U.S. National Comprehensive Cancer Network (NCCN). These guidelines advocate for anticancer therapies that incorporate 5-fluorouracil (5-FU) or gemcitabine, participation in clinical trials, or conservative management, contingent upon the systemic health of the CCA patient. 5-FU exerts its cytotoxic effects through the inhibition of the nucleotide synthesis enzyme thymidylate synthase (TS) and is utilized in both first- and second-line treatment protocols [Song et al. 2022]. Gemcitabine, particularly when combined with platinum-based agents, is acknowledged as the first-line chemotherapy regimen for intrahepatic cholangiocarcinoma (ICC). Nevertheless, the overall response rates to this treatment remain suboptimal [Valle et al. 2010; Sasaki et al. 2021]. There exists a notable deficiency of second-line treatment options for the majority of patients, as well as inadequate efficacy of postoperative chemotherapy in individuals diagnosed with CCA [Song et al. 2022; Rogers et al. 2014]. Given the unresolved mechanisms that underlie this disease and the scarcity of effective treatment modalities, there is an urgent need to identify biomarkers and therapeutic targets for the prognosis and management of CCA [Rodrigues et al. 2021; Duangkumpha et al. 2019]. Therefore, a personalized approach that takes into account the risk of recurrence and the overall health status of the patient is of paramount importance.

Zinc-α2-glycoprotein (AZGP1, ZAG) was first identified and purified from human serum in 1961 [Burgi et al. 1961]. This secretory protein is present in various bodily fluids, normal exocrine glandular epithelia across different tissues, adipose tissue, as well as in benign diseases and malignancies. In normal tissues, AZGP1 is associated with an increased susceptibility to obesity and the differentiation of adipocytes [Hassan et al. 2008; Hirai et al. 1998; Rolli et al. 2007]. In cancer, elevated levels of AZGP1 have been observed in the urine of patients suffering from cancer cachexia [Hirai et al. 1998; Sorensen-Zender et al. 2013, Bing et al. 2004]. Furthermore, AZGP1 has been reported to exhibit controversial roles, acting either as a tumor suppressor [Wen et al. 2024] or as an oncogenic [Fang et al. 2022; Liu et al. 2018] factor in various solid tumors. Given the multifaceted characteristics of AZGP1, it is imperative to investigate its biological functions not only in relation to its potential as a molecular target but also regarding its viability as a soluble biomarker. Programmed death Ligand 1 (PD-L1), also known as B7 homolog 1 and CD274, is a type I transmembrane glycoprotein that plays a crucial role in the establishment and maintenance of immunological self-tolerance by inhibiting the activity and proliferation of T-lymphocytes [Sharpe et al. 2007]. PD-L1 expression has been observed on the surfaces of various cancer cell types [Wang et al. 2016], including those originating from the kidney [Moller et al. 2021], ovary [Alwosaibai et al. 2023], and bladder [Eckstein et al. 2019]. Emerging studies are beginning to explore the tumor-intrinsic role of PD-L1 in promoting cancer initiation [Eckstein et al. 2019], metastasis [Guo et al. 2016; Vathiotis et al. 2021], and progression [Vathiotis et al. 2021; Sabatier et al. 2014]. Nevertheless, the specific role of membrane-bound PD-L1 in regulating cell death in CCA and the associated mechanisms have yet to be elucidated.

Despite ongoing research aimed at developing effective treatment modalities for cholangiocarcinoma, numerous challenges remain in the therapeutic landscape. Here, we elucidate the mechanism of cell death associated with the increased expression of soluble AZGP1 and report its potential as a predictive biomarker for therapeutic response to 5-FU treatment, as well as an immune activator in enhancing the efficacy of anti-cancer therapies. These findings could contribute to the development of more effective treatment strategies for cholangiocarcinoma.

## Materials and methods

### Cell culture and reagents

Human cholangiocarcinoma cell lines, KKU-213 (CVCL_M261), SNU-1196 (CVCL_5015), TFK-1 (CVCL_2214), SNU-308 (CVCL_5048), SNU-478, human embryonic kidney HEK293T (CVCL_3216) were obtained from American Type Culture Collection (ATCC, Manasssa, CA, USA), Korean Cell Line Bank (KCLB, Seoul, Korea) or Japanese Cancer Research Resources Bank (JCRB, Osaka, Japan). The cells were cultured in Dulbecco’s modified Eagle medium (DMEM) or RPMI-1640 medium (WELGENE, Daegu, Korea) supplemented with 10% fetal bovine serum (FBS, Giobco), 100 U/ml penicillin and 100 µg/ml streptomycin. All cells were incubated at 37 ℃ in humidified atmosphere containing 5% CO2. 5-flouracil (Selleckchem) was dissolved in optimal solvents and store at −20 ℃.

### Trypan blue assay

Cultured cells were prepared as single-cell suspensions by trypsin. Cells were dissociated by pipetting and mixed with an equal amount of 0.4% trypan blue (Gibco BRL). Stained cells were then counted under a light microscope (Olympus, Tokyo, Japan). Assays were repeated at least three times.

### CCA surgical mouse model

Five-week-old female BALB/c nude mice were purchased from GEM Biosciences. All experiments complied with animal protocols that had been approved by the Asan Medical Center Institutional Animal Care and Use Committee. In seven-week-old female BALB/c nude mice, a midline abdominal skin and muscle incision was performed to expose the xiphoid process for the establishment of a CCA surgical mouse model [Yang et al. 2011]. The hepatic bile duct was carefully isolated using microdissection forceps and then Ligated with a 4− 0 silk suture. The surgical protocol followed was based on previously published methods. Mice were sacrificed 9 days after surgery.

### Immunohistochemistry (IHC)

Formalin-fixed, paraffin-embedded tissue samples were sectioned into 5 μm slices, which were deparaffinized in xylene, rehydrated and washed with 0.1% TBST, and antigens were retrieved by boiling in target retrieval solution (Dako, CA, USA) for 20 min. Endogenous peroxidase activity was blocked using Dako Cytomatin Peroxidase Blocking Reagent (Dako) for 15 min, and tissue sections were maintained in Serum-Free Protein Block (Dako) at room temperature for 1 h. The antibodies used for immunohistochemistry were as follows: anti-AZGP1 (Abcam, ab180574, 1:200), anti-CA19-9 (ABclonal, A17437, 1:100), and anti-Ki67 (Abcam, ab16667, 1:200). Slides were stained with DAB chromogen (Vector Laboratories) according to the manufacture’s protocol and counterstained by hematoxylin mounting with histomount and scanning at ×20 magnification using a microscope.

### ShRNA and plasmids

The short hairpin RNA (shRNA) sequences targeting AZGP1 and PD-L1 (shAZGP1 and shPD-L1) were constructed by Genolution (Seoul, Korea). Cells were transfected with shAZGP1 or shPD-L1 using Lipofectamine RNAiMAX reagent (Invitrogen, #13778150) according to the standard protocol for 24 h and then treated 5-FU. The specific shRNA sequences used are as below: shAZGP1 (5’- CCAAGAUGGUCGUUACUCUUCUCAGAGUAACGACCAUCUUGGUU-3’). shPD-L1 (5’-CCUACUGGCAUUUGCUGAACGCAUUUCUCAAUGCGUUCAGCAAAUGCCAGUAGGUU-3’) PD-L1-pCMV-Myc-His-B was purchased from Sino Biological (Beijing, China) AZGP1-SPORT6 and Foxo1-Flag expression plasmid were purchased from Addgene. All plasmids were sequenced to confirm the absence of unwanted mutations.

### Quantitative reverse transcription polymerase chain reaction (RT-PCR)

Total RNA was extracted with TRIzol reagent (Invitrogen, Carlsbad, CA, USA) following the procedures provided by the manufacturer. The extracted RNA (1µg) was reverse transcribed using the AccuPower RT Premix (Bionner, Daejeon, Korea) in a Takara thermal cycler (Takara, China). cDNAs were synthesized using AccuPower RT Premix. PCR was done using primers for AZGP1 (forward 5‘-CCCAGATAACCAAGCAGAAG-3’, Reverse 5‘-CTAGCTGGCCTCCCA-3’), PD-L1 (forward 5‘- GTGAAAGTCAATGCCCCA-3’, Reverse 5‘-GCTAGCTTACGTCTCCTCCAAATGTGT-3’) and Foxo1 (forward 5‘-GTTTTCCAAATGGCCTGCAAG-3’, Reverse 5‘CCAGGCGCACAGTTATACT-3’). The expression of human glyceraldehyde-3-phosphate dehydrogenase (GAPDH) was determined in each sample and used as an internal control. Expression of Foxo1 and AZGP1 mRNA was compared on the basis of equivalent GAPDH transcript.

### Western blotting

To achieve AZGP1 overexpression, cells were cultured and subsequently transfected with pCMV-SPORT6-AZGP1 using Lipofectamine 2000 reagent (Invitrogen, #11668-019) according to the standard protocol for 24 h. For the treatment with conditioned media, cholangiocarcinoma (CCA) cells were maintained in DMEM or RPMI media supplemented with 10% fetal bovine serum (FBS), along with 100 U/ml penicillin and 100 µg/ml streptomycin. Following the incubation period, the CCA cells were harvested and subjected to centrifugation at 1500 g for 10 min. For co-immunoprecipitation (co-IP) experiments, constructs encoding the full-length PD-L1, PD-L1 domain 1 (amino acids 133–297), and PD-L1 domain 2 (amino acids 239–297) with Myc tag and constructs for full-length AZGP1, AZGP1 domain 1 (amino acids 21–205), and AZGP1 domain 2 (amino acids 204–298) with HA tag were created. Cells were lysed in RIPA buffer on ice. Total protein was electrophoresed by 8%−15% sodium dodecyl sulfate-polyacrylamide gel electrophoresis (SDS-PAGE) and transferred to polyvinylidene difluoride (PVDF) membranes for incubation with primary antibodies. Then, the membranes were incubated with HRP-conjugated secondary antibodies and protein bands detected were using a chemiluminescence western blotting detection kit (GE Healthcare Bio-Science, Sweden). The primary antibodies used in this study were as follows: AZGP1 (Abcam, ab180574, 1:2000), β-Actin (Santacruz, SC-47778, 1:3000), caspase-3 (Cell Signaling Technology, 9662 s, 1:1000), cleaved caspase-3 (Cell Signaling Technology, 9664 s, 1:1000), HA (Cell Signaling Technology, 3724 s, 1:1000), Ki-67, Foxo1 (Cell Signaling Technology, 2880 s, 1:1000), phospho-Foxo1 (Cell Signaling Technology, 9461 s, 1:1000), AKT (Cell Signaling Technology, 9272 s, 1:1000), phospho-AKT(Cell Signaling Technology, 4060 s, 1:1000), PD-L1 (Abcam, ab205921, 1:1000) Myc (Cell Signaling Technology, 2276 s, 1:1000).

### Extracellular AZGP1 enrichment and ELISA

The conditioned medium was concentrated with Amicon Ultra-2 Centrifugal Filter (Millipore Sigma) and centrifuged at 3,500 g for 30 min at 4 °C. To measure sAZGP1 in supernatant or whole blood samples, 96-well plated (SPL) were coated with a goat anti-AZGP1 polyclonal antibody (Lsbio) at 1 µg/ml. Bound sAZGP1 was detected using a rabbit anti-AZGP1 polyclonal antibody or a rabbit anti-AZGP1 monoclonal antibody. Absorbance was measured at 450 nm using a microplate reader (Bio-Rad).

### Immunofluorescence (IF)

The cells were cleaned with PBS and fixed using 4% paraformaldehyde for 10 min at room temperature. After rinsing the samples with 1 x PBS, 5% goat serum was added to block non-specific antibody binding sites and a primary antibody, fluorescent secondary antibody, and DAPI were added to label the target protein and cell nuclei. Finally, fluorescence images were viewed by a confocal microscope system (Zeiss LSM 780, Germany), and the results of co-localized pixels were analyzed using LSM version software (Zeiss, Germany).

### Flow cytometry for membrane protein expression

PBMCs from healthy donors were purchased in STEMCELL (Vancouver, Canada). Stimulated PBMCs were harvested and resuspended in FACS buffer (2% FBS and 0.05% sodium azide in PBS). Then, cells were first incubated with Human Fc block (BD Biosciences, #564219) for 30 min and stained with cocktail of anti-human cell surface marker antibodies, including anti-human CD45 (BD Biosciences, cat. no. 564357, RRID: AB_2744404), anti-human CD4 (BD Biosciences, cat. no. 557922, RRID: AB_396943), anti-human CD8 (BD Biosciences, cat. no. 563676, RRID: AB_2744463) at room temperature for 1 h. After that, cells were washed again, incubated with secondary antibody in the dark at room temperature for 30 min. Finally, the cells were washed again and measured by Flow cytometer (BD Biosciences, CA, USA). The data was analyzed using FACS Flow jo software.

### Statistical analysis

P values of less than 0.05 were statistically significant. Differences between two groups were estimated using t tests. Differences among four groups were evaluated using ANOVA with Tukey’s multiple comparison tests. All *p* < 0.01 or 0.05 were considered statistically significant.

## Results

### Soluble AZGP1 promotes CCA cell death

**Fig. 1:**
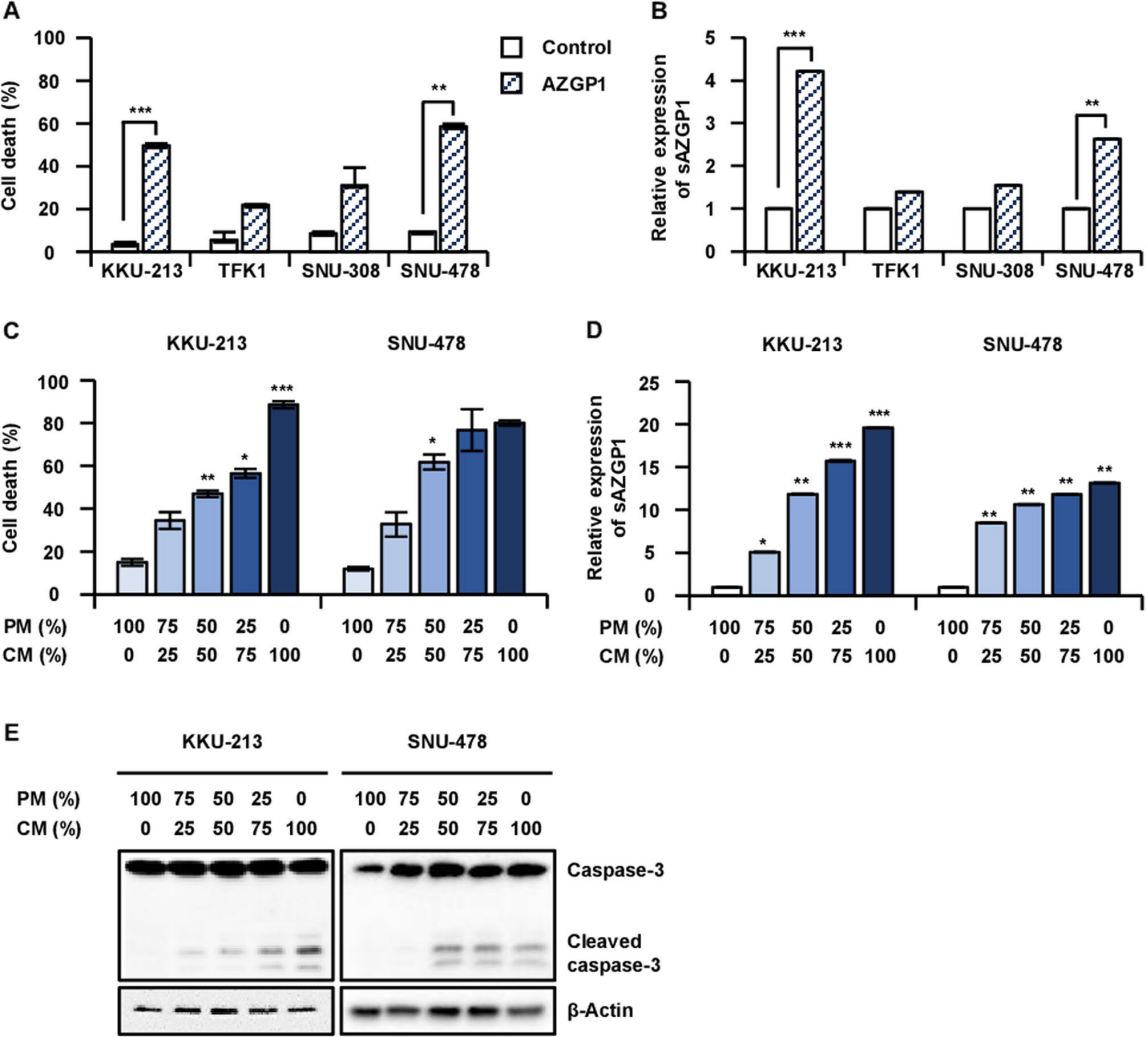
Soluble AZGP1 facilitates the cell death of CCA cells.** A** Cell death was confirmed through trypan blue assay after 36 h of AZGP1-overexpressing in human CCA cell lines. **B** ELISA was measured on secretion levels of AZGP1 from AZGP1 OE in CCA cell lines. **C** Cell death quantification was performed after treatment with AZGP1-OE CM in KKU-213 and SNU-478 cell at varying concentrations for 72 h. **D** The presence of sAZGP1 in the AZGP1-OE CM was confirmed through ELISA analysis. **E** Immunoblot analysis assessing level of the indicated proteins in KKU-213 and SNU-478. *P < 0.05, **P < 0.01, ***P < 0.001

In our previous study [Yun et al. 2024], we observed that AZGP1 is downregulated in human CCA cell lines. Furthermore, we established that the overexpression of AZGP1 induces cell death. To investigate the function of soluble AZGP1 (sAZGP1) in CCA, we performed experiments aimed at overexpressing AZGP1, which confirmed its secretion into conditioned media (CM). Notably, in most CCA cell lines, both cell death and the secretion of AZGP1 were significantly elevated following the overexpression of AZGP1 (Fig. 1A-B). To further explore the influence of the secretome derived from AZGP1-overexpressing CCA cells on cancer cell death human CCA cell lines were subjected to treatment with escalating concentrations of CM from these AZGP1-overexpressing cells. Cell death was quantified using the trypan blue assay, while the presence of sAZGP1 in the CM was measured via ELISA (Fig. 1C-D). The results suggest that sAZGP1 induces cell death (Fig. 1E), with this effect becoming more pronounced at higher concentrations.

### sAZGP1 is downregulated in the CCA mice model

**Fig. 2:**
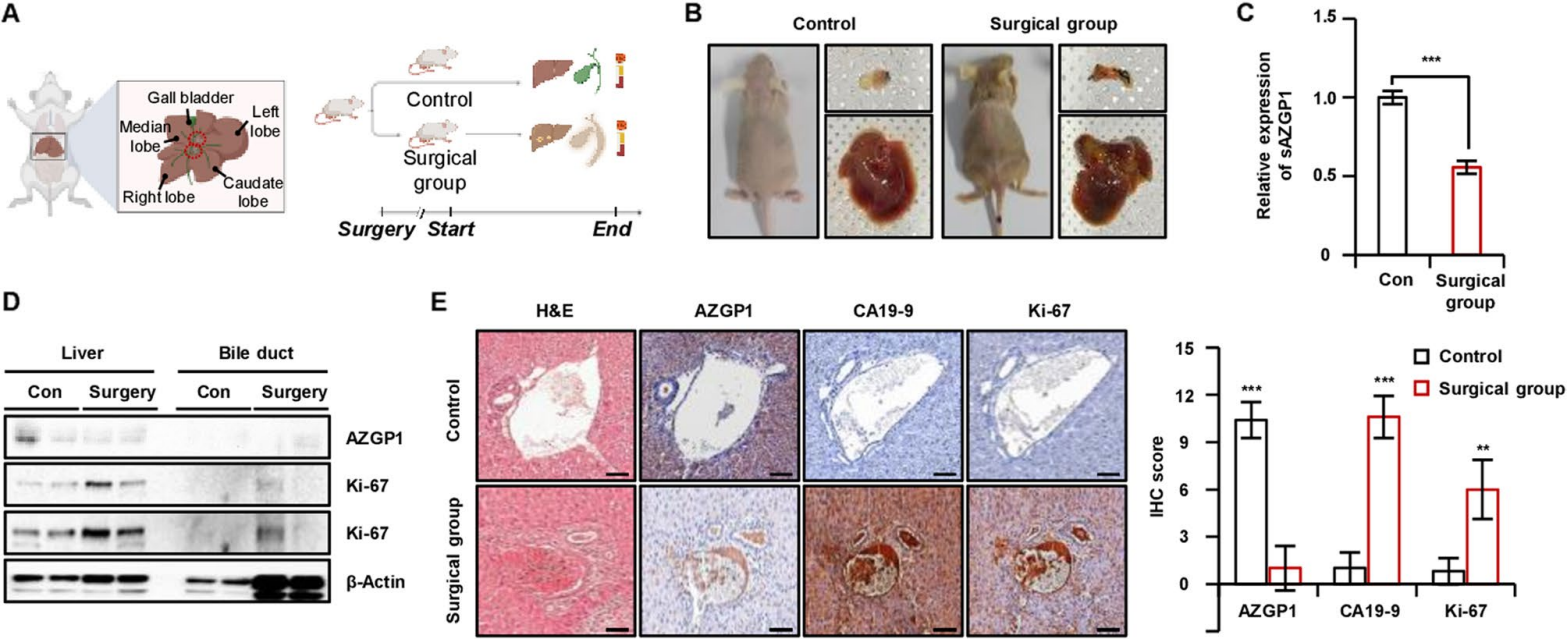
In the CCA mouse model, the expression of sAZGP1 is significantly reduced.** A** The schematic illustrates the experimental design. A surgical procedure was performed to establish a mouse model of CCA in BALB/C mice (*n* = 5 mice, per groups) **B** Representative images of depict the whole body of control and surgical mice (Left), the biliary duct (Top Right) and the liver (Bottom Right) at the study endpoint. **C** The quantification of sAZGP1 in the blood was conducted through ELISA. **D** The expression levels of AZGP1 and Ki-67 were assessed via western blot analysis in Liver and bile duct tissues. Tumors were collected 9 d post- surgery (*n* = 5 mice per group). **E** Representative immunohistochemical (IHC) staining for AZGP1, CA19-9 (a CCA marker), and Ki-67 in bile duct tissues from control and surgical group mice (Left panel). Quantification of IHC staining was performed showing reduced AZGP1 and increased CA19-9 and Ki-67 expression in the surgical group (Right panel). Scale bar = 50 μm. **P < 0.01, ***P < 0.001

The transformation of bile duct epithelial cells into malignant cells predominantly occurs in the context of chronic cholestasis and inflammatory conditions [Banales et al, 2020]. This persistent stimulation of the cells creates an environment that promotes malignancy, ultimately contributing to the pathogenesis of CCA. To investigate the presence of AZGP1 in CCA as a soluble form, we established a CCA-induced mouse model in BALB/c mice [Yang et al. 2011] (Fig. 2A). Jaundice, edema and abdominal distension were observed in the CCA-induced model mice in contrast to the control (healthy) mice, and autopsies revealed bile duct hypertrophy along with multiple nodules with various sizes throughout the liver (Fig. 2B). Blood plasma and tumor tissue were collected from the surgical mice at the study endpoint to assess whether tumor formation could lead to a reduction in plasma levels of AZGP1 and/or alter the expression of specific proteins. Histological analysis of the surgical mice demonstrated additional malignancy indicators, including necrosis and regions of moderately differentiated tumors. Compared to the control group, we confirmed a significant overexpression of CCA markers CA19-9 and Ki-67, while AZGP1 exhibited markedly lower expression levels in the surgical group (Fig. 2D-E). Furthermore, the concentration of sAZGP1 in the plasma of the mice was significantly reduced compared to that of healthy mice Fig. 2C.

### Upregulation of Foxo1 regulates AZGP1 transcription and secretion

**Fig. 3:**
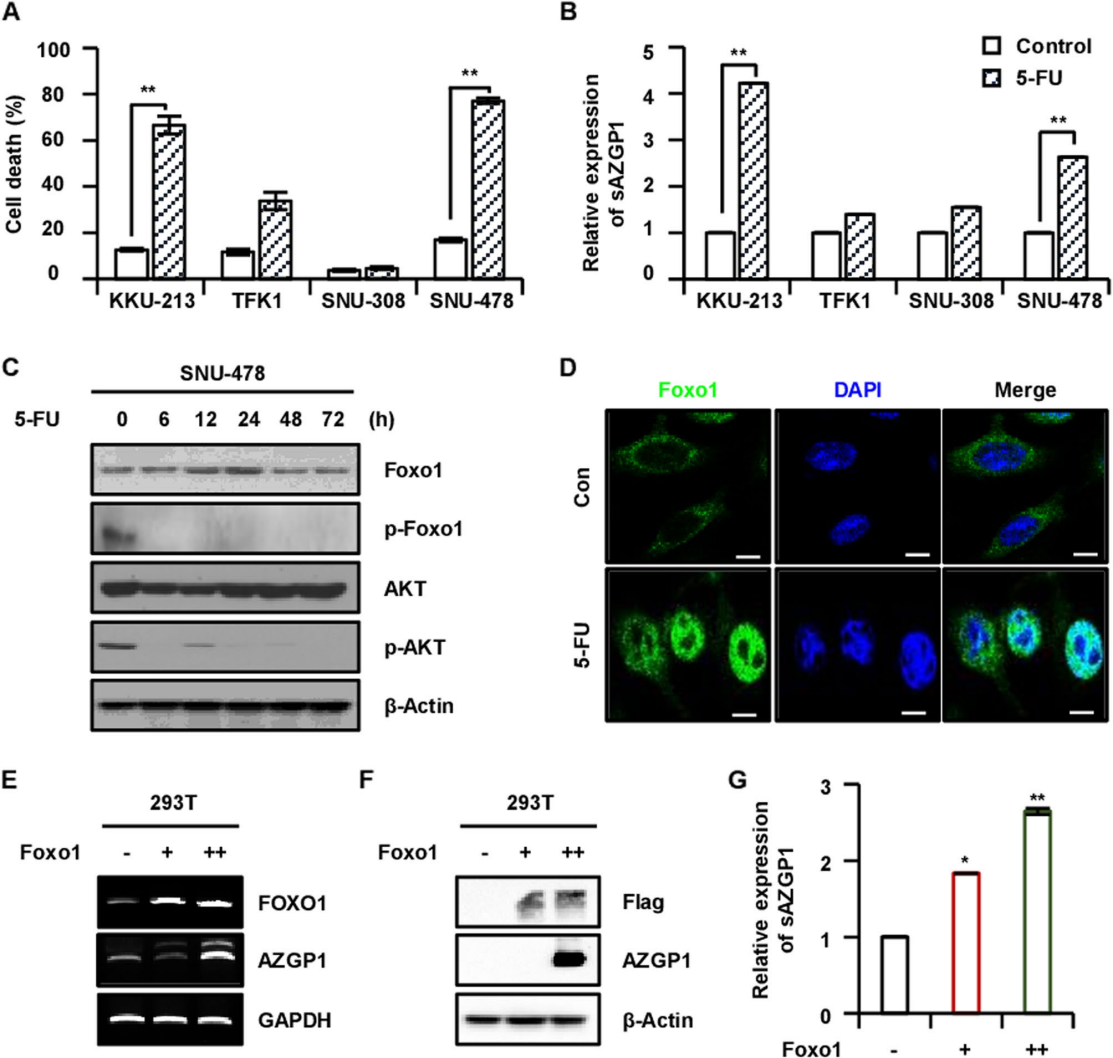
The upregulation of Foxo1 influences the transcription and secretion of AZGP1.** A** Cell death was quantified in CCA cells treated with 5-FU at a concentration of 10 µM for 72 h. **B** ELISA was conducted to measure the expression of sAZGP1 in CCA cells treated to 5-FU at 10 µM for 72 h. **C** Western blot analysis was performed on CCA cells following treatment with 5-FU at 10 µM for 72 h. **D** Western blot analysis of SNU-478 cells revealed time-dependent changes in the expression of p-Foxo1 and p-AKT after treatment with 5-FU. **E** Immunofluorescence was utilized to assess the localization of Foxo1 in SNU-478 cells, comparing non-treated controls (Con) with those treated with 5-FU at 10 µM for 24 h (5-FU). (Scale bar: 50 μm). Additionally, the mRNA **F** and protein levels **G** of AZGP1 were confirmed in relation to the overexpression of Foxo1. *P < 0.05, **P < 0.01

Next, we investigated the cell death of CCA and the expression levels of sAZGP1 that were induced by 5-FU treatment. Following treatment with 5-FU, an increase in cell death was observed across all cell lines, with the exception of SNU-308 (Fig. 3A) and the expression of sAZGP1 was significantly elevated (Fig. 3B). Notably, the KKU-213 and SNU-478 cell lines demonstrated the highest levels of both cell death and sAZGP1 expression following treatment with 5-FU. The findings indicate that the soluble form of AZGP1 is expressed at reduced levels in CCA, and that its expression can be enhanced following treatment with 5-FU. Subsequently, we aimed to elucidate the mechanism underlying the upregulation of AZGP1 in response to 5-FU. We hypothesized that Foxo1, a gene regulated by 5-FU [Li et al. 2013; Han et al. 2024], may be activated following treatment, leading to an increase in both the transcriptional and protein levels of AZGP1. Initially, we treated the SNU-478 cell Line with 5-FU in a time-dependent manner and observed a decrease in p-Foxo1 and p-AKT, suggesting that the AKT-Foxo1 signaling pathway is influenced by 5-FU (Fig. 3C). Furthermore, we sought to ascertain whether the Foxo1 activated by 5-FU translocated from the cytosol to the nucleus. This translocation was confirmed through IF, which demonstrated a significant nuclear accumulation of Foxo1 in CCA cells treated with 5-FU, compared to the control group (Fig. 3D). Furthermore, the upregulation of AZGP1 mRNA and protein levels, resulting from the overexpression of Foxo1 (Fig. 3E-G), provides support to our hypothesis.

### PD-L1 is a significant interaction partner of sAZGP1

**Fig. 4:**
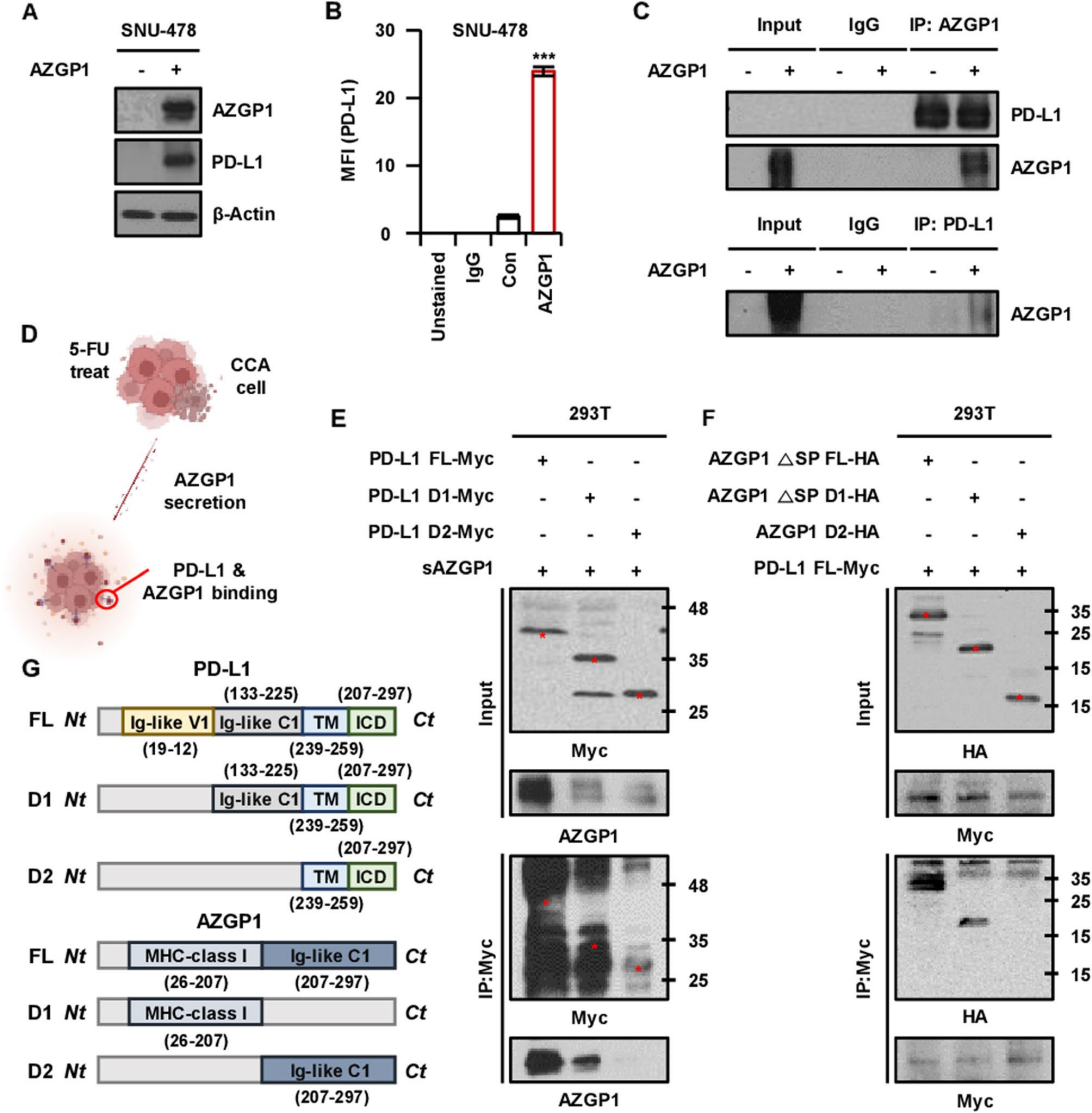
PD-L1 functions as a significant interaction partner of sAZGP1.** A** The expression of levels AZGP1 and PD-L1 in SNU-478 cells overexpressing AZGP1 for 36 h, as determined by western blot analysis. **B** Representative quantification of flow cytometry measuring PD-L1 surface expression in both control and AZGP1 overexpressing cells. **C** Co-immunoprecipitation results demonstrated the interaction between sAZGP1 and PD-L1 in SNU-478 cells 36 h following transfection. **D** A schematic of the functional domains of human AZGP1 and PD-L1. **E-F** Co-immunoprecipitation further indicated the interaction between AZGP1 △SP D1-HA and PD-L1 D1-Myc in 293 T cells, 24 h post-transfection **G** Illustrating the action of sAZGP1 secreted from CCA cell lines interacting with membrane PD-L1. **P < 0.01

To identify the cell surface binding receptors for AZGP1, we conducted an analysis of protein-protein interaction networks for AZGP1 using the BioGRID database (https://thebiogrid.org). Based on liquid chromatography-mass spectrometry (LC/MS) data [Meng et al. 2020] indicating that PD-L1 and AZGP1 may function as binding partners, we validated the potential of PD-L1 as a binding candidate for sAZGP1 through co-IP. The expression of membrane PD-L1 was observed to increase in correlation with the overexpression of AZGP1 (Fig. 4A-B). And then, after pulling down the sAZGP1 and PD-L1 complex, we confirmed that sAZGP1 interacts with membrane PD-L1 in CCA cells by detecting co-precipitated PD-L1 or AZGP1 through Western blotting (Fig. 4C). To ascertain the specific PD-L1 domain that interacts with sAZGP1, we constructed AZGP1 and PD-L1 domain constructs (Fig. 4G). Myc-tagged PD-L1 subdomains were transfected into 293 T cells, leading to the expression of PD-L1 FL (full-length PD-L1), Domain 1 (Ig-like C1 + transmembrane (TM) + intracellular domain (ICD), and Domain 2 (TM + ICD). An anti-AZGP1 antibody was employed to immunoprecipitated PD-L1 from the lysates of the complex (sAZGP1 + PD-L1 domains), followed by Western blot analysis to detect PD-L1. This analysis revealed the co-IP of AZGP1 Domain 1 with PD-L1 Domain 1 (Fig. 4E-F). Collectively, these findings suggest that sAZGP1 interacts with the extracellular domain of PD-L1 (Fig. 4D).

### The Blockade sAZGP1 and PD-L1 suppresses cell death in CCA cells

**Fig. 5:**
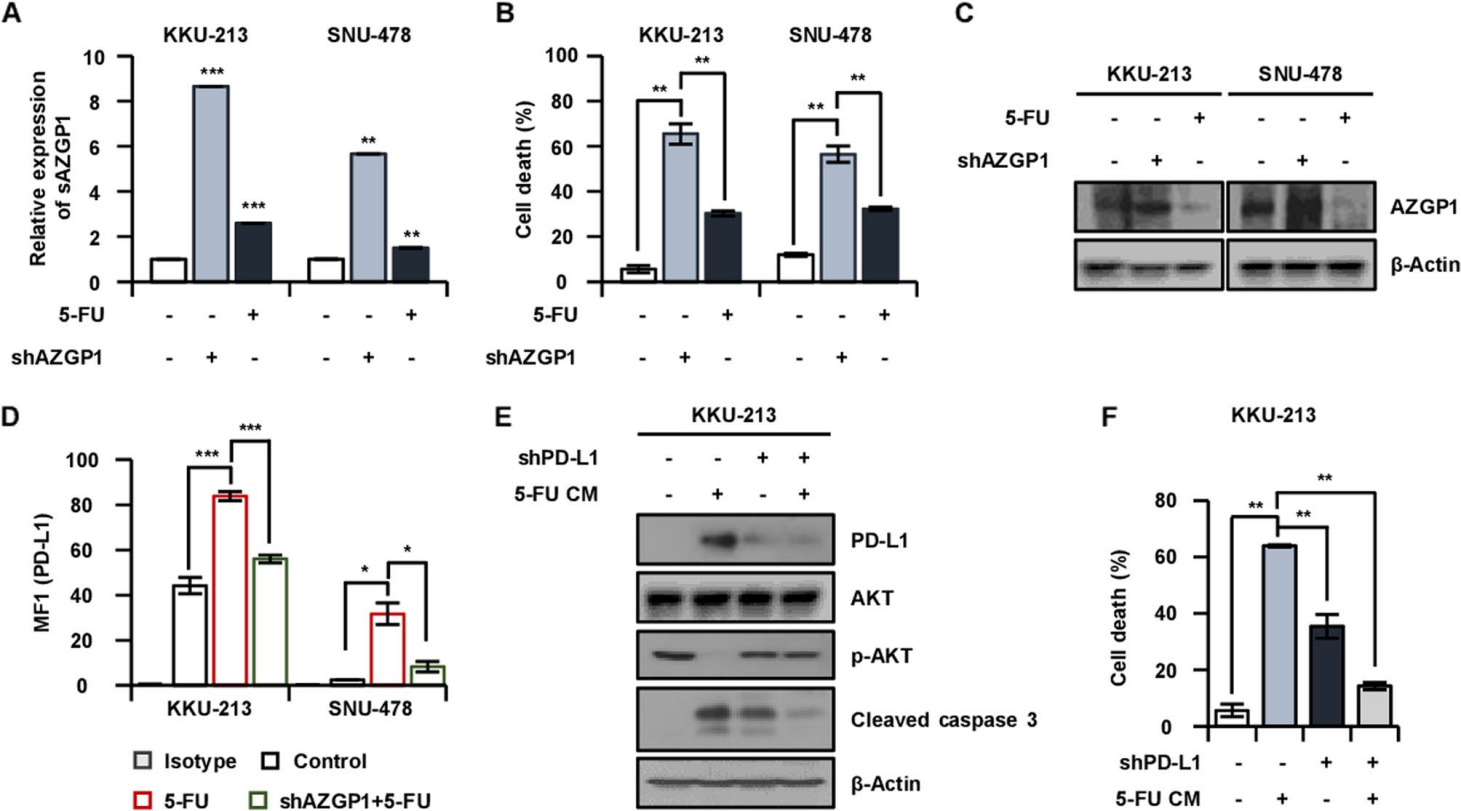
The inhibition of sAZGP1 and PD-L1 results in the attenuation of cell death in CCA cells.** A** After shAZGP1 transfection for 24 h in KKU-213 and SNU-478 cell lines, the expression level of sAZGP1 was confirmed after treatment with 10 µM of 5-FU. **B** Reduced cell death was confirmed following shAZGP1 and 5-FU treatment. **C** The reduced expression of AZGP1 and PD-L1 following shAZGP1 and 5-FU treatment was confirmed by western blot. **D** The reduced expression of membrane PD-L1 following shAZGP1 and 5-FU treatment was confirmed by FACS. **E** Changes in p-AKT and cleaved caspase 3 were confirmed by western blot after shPD-L1 transfection for 24 h followed by 5-FU CM treatment. **F** Cell death was confirmed following shPD-L1 transfection and 5-FU CM treatment. **P < 0.01, ***P < 0.001

The time-dependent treatment of 5-FU induces AZGP1 secretion and cell death (Fig. S1A-B), and it has also been confirmed that a significant increase of PD-L1 in both the mean fluorescence intensity (MFI) and protein level in the 5-FU-treated CCA cells compared to the control (Fig. S1C-D). To elucidate the role of AZGP1 secreted from CCA cells treated with 5-FU in relation to cell death, we confirmed cell death through a CM percentage-dependent treatment. Our results demonstrated an increase in AZGP1 expression that was dependent on the CM percentage (Fig. S2A). As the CM percentage increased, both cell death and membrane PD-L1 expression exhibited a corresponding increase (Fig. S2B-C). These findings led to the hypothesis that sAZGP1 may promote cell death by activating membrane PD-L1. PD-L1 is known to regulate several downstream signaling pathways [Cha et al. 2019], including the PI3K/AKT [Guo et al. 2024; Peng et al. 2022], MAPK/ERK1/2 [Peng et al. 2022; Gao et al. 2022], and mammalian target of mTOR [Cao et al. 2023] pathways. Consequently, we further investigated whether these keys signaling pathways are modulated by PD-L1 expressed on tumor cells. Our results indicated a decrease in p-AKT expression alongside an increase in cleaved caspase 3 expression in a CM percentage-dependent manner (Fig. S2D). This suggests that the AZGP1 secreted in the CM from 5-FU-treated cells is sufficient to induce AKT activation, which is mediated by membrane PD-L1. We posited that the interaction between sAZGP1 and PD-L1 could boost a synergistic effect on anticancer activity along with the anticancer effects of 5-FU. To underscore the significance of the interaction between these two targets, we downregulated the expression of each gene to assess the resultant changes in cell death and associated pathways. To investigate the impact of sAZGP1 on cell death, we silenced AZGP1 and subsequently treated the CCA cell Line with 5-FU and observed a significant reduction of both endogenous and exogenous AZGP1 protein levels and cell death (Fig. 5A-C). Furthermore, the depletion of PD-L1 resulted in an upregulation of p-AKT protein levels, which ultimately influenced cell death (Fig. 5D-F). Collectively, our findings demonstrate that AZGP1 is specifically expressed in a soluble form by CCA cells treated with 5-FU, which may influence the cell death through the activation of the PD-L1-dependent AKT signaling pathway.

### sAZGP1 affects T-cell activation in CCA microenvironment

**Fig. 6:**
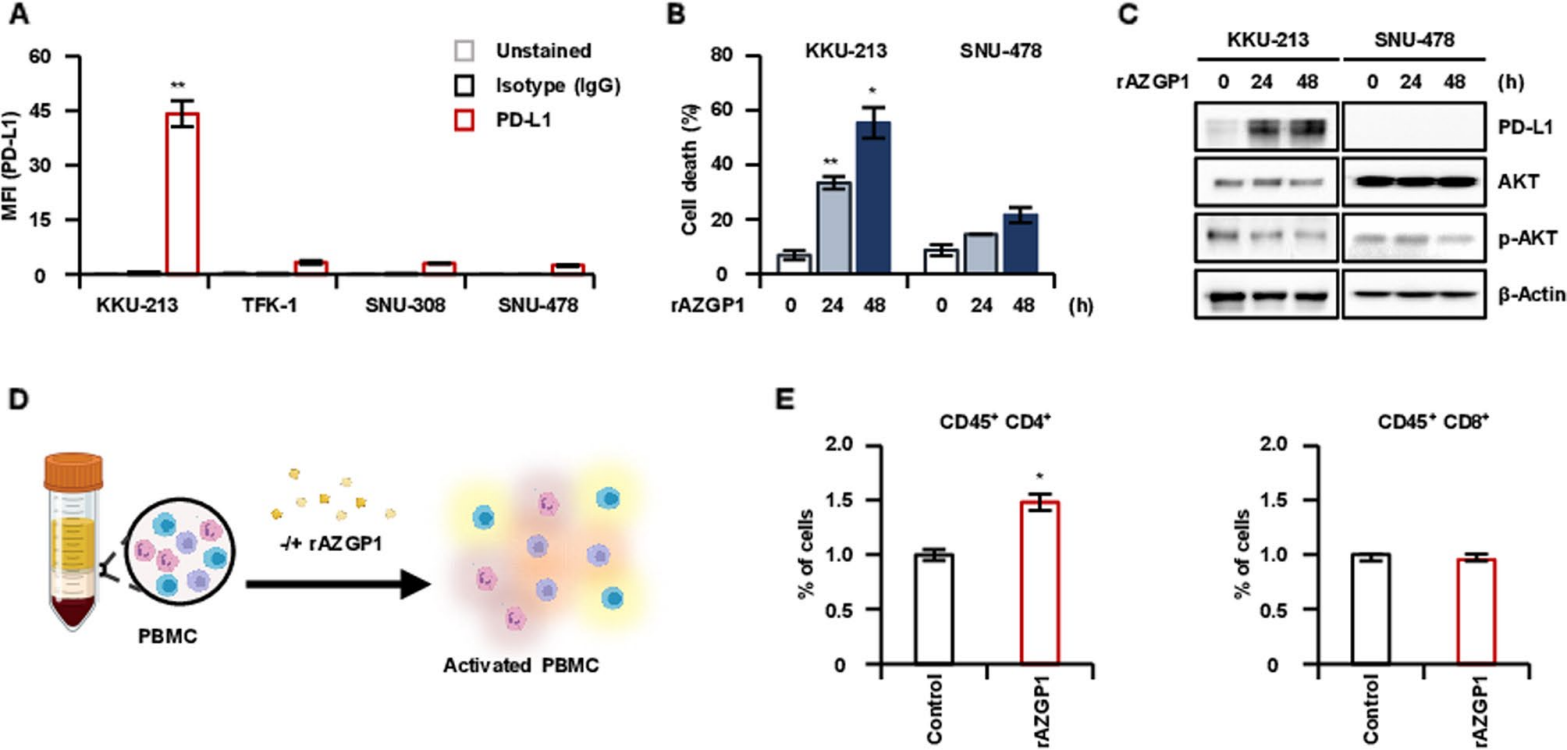
sAZGP1 influences T-cell activation within the microenvironment of CCA. **A** The expression of membrane PD-L1 in CCA cell lines was confirmed through FACS. **B** Cell death was quantified following treatment with recombinant AZGP1 in cell lines. **C** Changes in p-AKT following recombinant AZGP1 treatment were validated using western blot analysis. **D** A schematic representation of the experimental design was provided to confirm the activation of immune cells in peripheral blood mononuclear cells (PBMC) following recombinant AZGP1 treatment. **E** FACS of activated immune cells expressing CD45^+^CD4^+^ and CD45^+^CD8^+^ markers in PMBC following treatment with recombinant AZGP1. **F** A schematic depiction illustrates that the upregulation of sAZGP1 enhances the synergistic anticancer effects of PD-L1-mediated cell death and the activation of CD4 ^+^ T cells. *P < 0.05, **P < 0.01, ***P < 0.001

Through the demonstration of a reduction in cell death associated with the inhibition of PD-L1 expression, we highlighted the essential role of membrane PD-L1 in the cell death mechanism mediated by sAZGP1 (Fig. 5). To elucidate the relationship between sAZGP1 and the expression of membrane PD-L1 in CCA cell lines, we evaluated the levels of membrane PD-L1 across various human CCA cell lines. The KKU-213 cell line exhibited a PD-L1 positivity of 44.2%, while the SNU-478 cell Line demonstrated the lowest expression at 2.4% (Fig. 6A). Subsequently, we treated two CCA cell Lines with 0.5 µg/ml of human recombinant AZGP1 protein. Notably, treatment with recombinant AZGP1 led to a significant induction in cell death in the KKU-213 cell line, whereas no such effect was noted in the SNU-478 cell line (Fig. 6B). Following stimulation with AZGP1, we observed a reduction in p-AKT levels in both PD-L1 high and PD-L1 low CCA cell lines, with a more pronounced effect observed in the PD-L1 high KKU-213 cell line (Fig. 6C). Subsequently, we identified a variety of immune cell types activated by sAZGP1 utilizing peripheral blood mononuclear cells (PBMCs) obtained from healthy donors (*n* = 3) (Fig. 6D). As shown in Fig. 6e, treatment with recombinant AZGP1 in PBMCs resulted in a significant increase in the population of CD4^+^ T cells, while CD8^+^ T cells exhibited a comparatively slightly increase relative to CD4^+^ T cells. These findings suggest that secreted AZGP1 not only induces apoptosis in CCA cells but also activates immune cells, particularly CD4^+^ T cells, thereby enhancing anti-cancer effects.

## Discussion

Recent studies have indicated that AZGP1 is downregulated in CCA compared to normal tissues, which correlates with overall survival and patient prognosis [Yun et al. 2024; Deng et al. 2023]. CCA ranks as the second most common primary liver malignancy, following hepatocellular carcinoma, and can manifest at any site within the biliary tract. The disease’s insidious onset, combined with its aggressive nature and persistent resistance to chemotherapy, results in a high mortality rate, contributing to approximately 2% of global cancer-related deaths annually. The importance of therapeutic biomarkers for the development of more effective treatment strategies is increasingly recognized. Recent advancements in liquid biopsy techniques, which are less invasive and more cost-effective than traditional biopsy methods, have attracted significant research interest. This study aims to explore soluble biomarkers as predictive indicators of chemotherapy efficacy. Recent investigations into the pathogenesis of CCA have identified several genes and proteins involved in its molecular development, leading to the proposal of various tumor markers based on these mechanisms [Kimawaha et al. 2021; Spencer et al. 2023]. Notably, AZGP1 has been shown to inhibit epithelial-mesenchymal transition (EMT) in tumor cells [Kong et al. 2010; Xu et al. 2016], suppress tumor growth, and promote apoptosis [Li et al. 2020]. Several studies have suggested that AZGP1 may serve as a diagnostic biomarker for colorectal [Ji et al. 2013] and gastrointestinal cancers [Huang et al. 2013]. Its role in various cancers has been characterized as either a tumor suppressor or an oncogene. Importantly, one study reported that AZGP1 expression in cancerous tissues was significantly lower than in adjacent non-cancerous tissues [Yun et al. 2024; Deng et al. 2023]. Furthermore, patients with ICC exhibiting elevated AZGP1 levels demonstrated improved overall survival (OS) compared to those with lower levels, as indicated by Kaplan-Meier survival curve analysis [Huang et al. 2013]. The potential of AZGP1 as a soluble biomarker has also been reported in the serum of patients with gastrointestinal tract cancers [Huang et al. 2013] and colorectal cancer [Ji et al. 2013]. However, the comprehensive role of sAZGP1 in cancer remains largely undefined, and the significance of sAZGP1 expression and its implications in CCA have yet to be elucidated.

The results of our study demonstrate that plasma levels of sAZGP1 in CCA mice models were significantly lower compared to those in healthy mice, suggesting that baseline sAZGP1 expression can effectively distinguish between CCA and normal plasma. Importantly, AZGP1, which is expressed at low levels in CCA, was found to be upregulated following treatment with 5-FU. This upregulation of AZGP1, both intracellularly and extracellularly, appears to be mediated by the AKT/Foxo1 signaling pathway. In CCA cell Lines, 5-FU treatment influences the mRNA and protein expression of AZGP1 through the translocation of Foxo1 into the nucleus. To elucidate the role of sAZGP1 further, we established the interaction between sAZGP1 and PD-L1. Domain analysis revealed that sAZGP1 binds to the extracellular domain of PD-L1. The cell death induced by treatment with CM was supported by observed changes in the AKT signaling pathway, which is one of the downstream signals of PD-L1. These findings suggest that AZGP1, secreted in response to 5-FU treatment in CCA, interacts with the extracellular domain of PD-L1, thereby activating the AKT signaling pathway and ultimately leading to cell death in CCA cell lines. Functionally, the depletion of AZGP1 resulted in reduced secretion of sAZGP1 and increased cell death in human CCA cell lines. Additionally, silencing of PD-L1 led to a decrease in PD-L1 protein levels while restoring cell viability in the context of 5-FU CM-mediated cytotoxicity. This indicates that the interaction between these target proteins is a critical factor in CCA cell death. We further confirmed the potential of sAZGP1 as an immune cell activator by identifying specific types of activated immune cells in PBMCs following recombinant AZGP1 treatment.

This observation suggests that sAZGP1 not only induces cell death as part of its anticancer mechanism but also plays a role in modulating immune responses, indicating a potential synergistic effect that may enhance anticancer efficacy.

## Conclusion

We elucidate the mechanism of cell death associated with the increased expression of soluble AZGP1 and report its potential as a predictive biomarker for therapeutic response to 5-fluorouracil treatment, as well as an immune activator in enhancing the efficacy of anti-cancer therapies.

## Supplementary Information

Supplementary Material 1.

Supplementary Material 2.

Supplementary Material 3.

## Data Availability

No datasets were generated or analysed during the current study.

## Abbreviations

CCA: Cholangiocarcinoma
5-FU: 5-fluorouracil
AZGP1: Zinc-α2-glyxoprotein
sAZGP1: Soluble Zinc-α2-glyxoprotein
PD-L1: Programmed death ligand1
AKT: Protein kinase B
phospho-AKT: Phopho protein kinase B
Foxo1: Forkhead Box O1
Phosphor Foxo1: Phospho Forkhead Box O1
GAPDH: Glyceraldehyde-3-phosphage dehydrogenase
β-Actin: Beta actin
CM: Conditioned media
IF: Immunofluorescence
IHC: Immunohistochemistry
FACS: Flow cytometry
ELISA: Enzyme-Linked Immunosorbent Assay
CA19-9: Carbohydrate antigen 19-9
CD45: Protein tyrosine phosphatase receptor type C
CD8: Cluster of differentiation 8
CD4: Cluster of differentiation 4
