# Supplementary figures and images for: Secreted AZGP1 induced by 5-FU binds to PD-L1 and promotes apoptosis in cholangiocarcinoma
Source: Mol Med. 2025 Sep 26;31:295. doi: 10.1186/s10020-025-01362-8 (PMC12465573; doi:10.1186/s10020-025-01362-8)

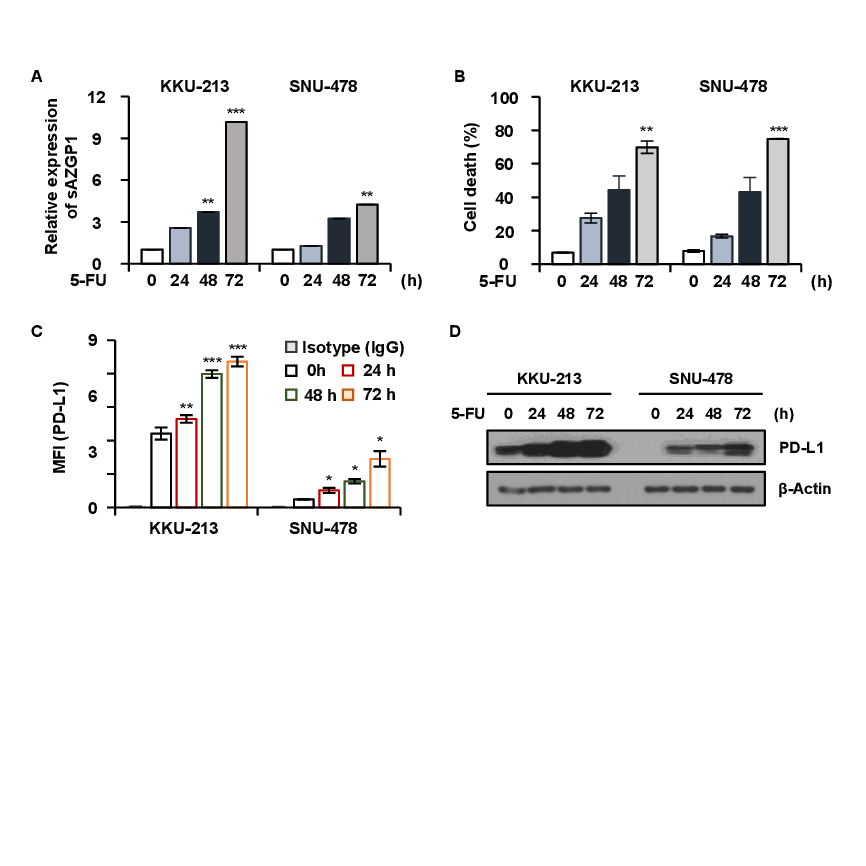

Supplement: Supplementary file 1 — Supplementary Material 1. [file 10020_2025_1362_MOESM1_ESM.tiff]

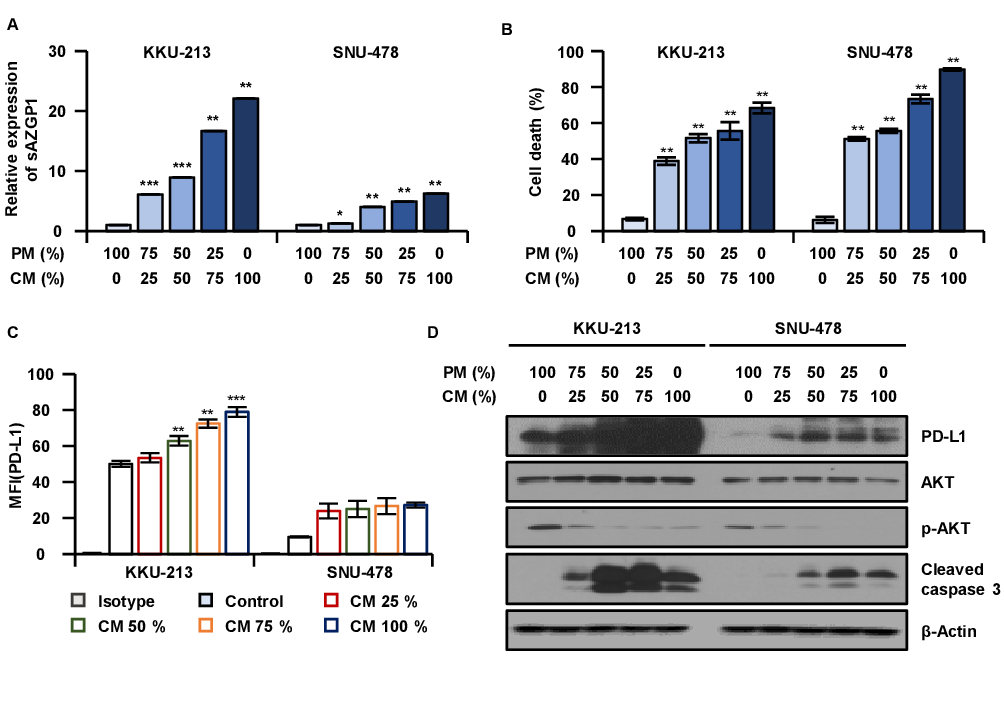

Supplement: Supplementary file 2 — Supplementary Material 2. [file 10020_2025_1362_MOESM2_ESM.tiff]

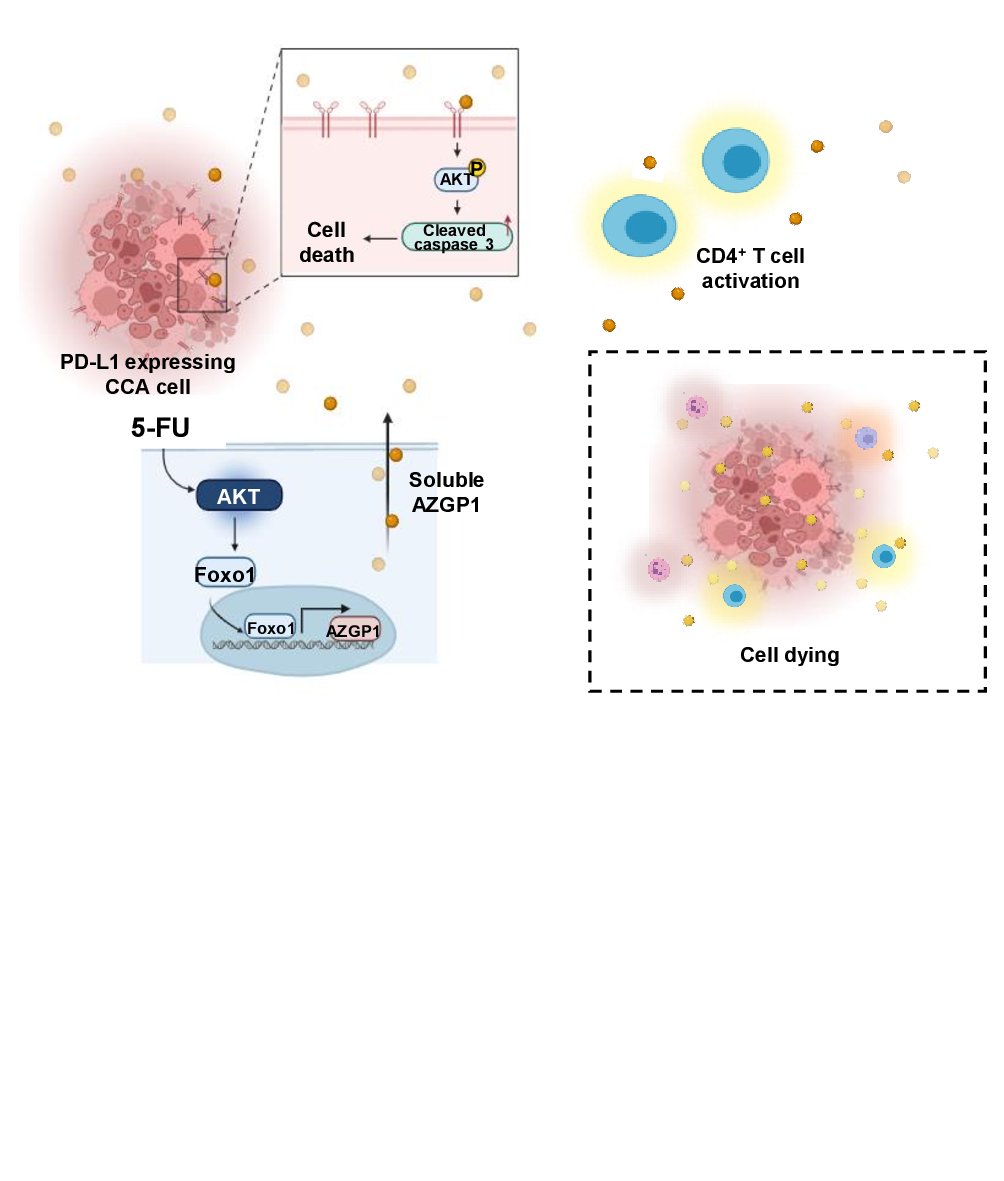

Supplement: Supplementary file 3 — Supplementary Material 3. [file 10020_2025_1362_MOESM3_ESM.tiff]
